# Scaling Down Large-Scale Thawing of Monoclonal Antibody Solutions: 3D Temperature Profiles, Changes in Concentration, and Density Gradients

**DOI:** 10.1007/s11095-021-03117-6

**Published:** 2021-11-02

**Authors:** Oliver Bluemel, Jakob W. Buecheler, Astrid Hauptmann, Georg Hoelzl, Karoline Bechtold-Peters, Wolfgang Friess

**Affiliations:** 1 Department of Pharmacy, Pharmaceutical Technology and Biopharmaceutics, Ludwig-Maximilians-Universitaet Muenchen, 81377 Munich, Germany; 2 Technical Research and Development, Novartis Pharma AG, 4002 Basel, Switzerland; 3 Sandoz GmbH, 6336 Langkampfen, Austria

**Keywords:** Concentration gradient, Freeze–thaw, Large-scale thawing, Monoclonal antibody, Scale-down device

## Abstract

**Purpose:**

Scale-down devices (SDD) are designed to simulate large-scale thawing of protein drug substance, but require only a fraction of the material. To evaluate the performance of a new SDD that aims to predict thawing in large-scale 2 L bottles, we characterised 3D temperature profiles and changes in concentration and density in comparison to 125 mL and 2 L bottles. Differences in diffusion between a monoclonal antibody (mAb) and histidine buffer after thawing were examined.

**Methods:**

Temperature profiles at six distinct positions were recorded with type T thermocouples. Size-exclusion chromatography allowed quantification of mAb and histidine. Polysorbate 80 was quantified using a fluorescent dye assay. In addition, the solution’s density at different locations in bottles and the SDD was identified.

**Results:**

The temperature profiles in the SDD and the large-scale 2 L bottle during thawing were similar. Significant concentration gradients were detected in the 2 L bottle leading to marked density gradients. The SDD slightly overestimated the dilution in the top region and the maximum concentrations at the bottom. Fast diffusion resulted in rapid equilibration of histidine.

**Conclusion:**

The innovative SDD allows a realistic characterisation and helps to understand thawing processes of mAb solutions in large-scale 2 L bottles. Only a fraction of material is needed to gain insights into the thawing behaviour that is associated with several possible detrimental limitations.

**Supplementary Information:**

The online version contains supplementary material available at 10.1007/s11095-021-03117-6.

## Introduction

Therapeutic proteins, especially monoclonal antibodies (mAbs), are of significant importance in a constantly growing market and number of indications [1–3]. During large-scale production of biopharmaceuticals freezing is a commonly used processing step to enhance physical and chemical stability and to increase shelf life [4, 5]. In comparison to liquid storage, frozen storage and transport offers flexibility for the manufacturing process by decoupling drug substance and drug product processing, decreased risk of microbial growth and reduced foaming, shaking and agitation [4–6]. While freezing and thawing (FT) is intentional during production of bulk drug substances and drug products [7], commercial products may also be frozen multiple times accidentally by mishandling. Despite its apparent simplicity and undoubted advantages, freezing can be associated with a variety of possible drawbacks. Cold denaturation [8], cryoconcentration on a microscopic and macroscopic scale [2, 3, 9], crystallisation of cryoprotectants [10, 11] or buffer salts, associated with significant pH shifts [12], interactions at the ice interface [13], and ultimately conformational and colloidal instability [14] are well known, although not fully understood. In addition, Authelin et al. address enhanced oxidation, the formation of air bubbles, local pressure, and mechanical stresses as further considerable occurrences that are so far not well examined [15]. The FT process becomes even more complex as the described phenomena are not only relevant during freezing but also during thawing. Especially crystallisation [7, 16] during and concentration gradients [17, 18] after thawing can lead to denaturation, aggregation and precipitation of proteins [18, 19].

During freezing, growing ice crystals exclude proteins and other excipients. Consequently, the concentration of the remaining unfrozen solution increases. The microenvironment of the protein between the ice crystals can be described as a freeze-concentrated matrix (FCM). Under ideal equilibrium conditions its maximum concentration is characterised by state diagrams [1, 15]. The glass transition temperature of the maximally freeze concentrated solution (T_g_′) is the temperature of complete solidification of the FCM. Macroscopically, the cryoconcentrated solution near the freezing front is transported away into unfrozen regions by diffusion and convection. This leads to a substantial heterogeneity throughout the frozen bulk [1, 20]. During the thawing process the FCM melts out of the frozen bulk leaving nearly pure ice behind. Due to the high density of the FCM and the low density of the ice, ice floats on top and dilutes the top region as it melts [19]. Several studies report a tremendous concentration gradient in post-thawed large-scale bottles [17–19]. These gradients can be associated with decreased protein stability. Protein precipitation can occur in the top layer due to lack of stabilisers or at the bottom because of a salting out effect [17, 19].

The complex and interdependent processes have been studied using small-scale experiments and specific scale-down devices (SDDs) [2, 6, 10, 14, 16, 21, 22]. Such experiments support finding of the optimal formulation, revealing limitations during processing and storage, and unveiling aggregation mechanisms under the chosen FT setup. However, the experiments do not reflect the large-scale FT rates, cryoconcentration, surface area of ice, and exposure time of protein to the numerous stresses and therefore cannot be straightly extrapolated to large-scale [1, 20, 23, 24].

Using Computational Fluid Dynamics (CFD) the heat transfer can be adapted so that mass fractions in small- and large-scale containers experience equivalent stress. Based on this approach SmartFreeZ developed a SDD assisted by CFD following a novel and innovative approach to representatively scale down FT behaviour in a 2 L bottle. The scale-down strategy by CFD has been described previously [24]. In contrast to matching FT profiles at the last point to freeze (LPTF) or the last point to thaw (LPTT), simulations were used to divide the bulk into control volumes of approximately 1 mm^3^. The time between the beginning of freezing in the container and reaching T_g_′ in the control volume can be calculated. This time span is most detrimental for protein molecules as they become exposed to the ice liquid interface and concentrated together with the other solutes. When passing T_g_′, the viscosity of the FCM increases considerably and the decreased mobility prevents aggregation [1, 11, 25]. The thermal history of a protein solution during large-scale FT was translated into the cumulative thermal history in the SDD. The SDD, which matches the thermal history of a large-scale 2 L bottle, consists of a 125 mL bottle surrounded by a unique holder that controls heat exchange.

Previously, we characterised the performance of this innovative SDD during freezing, revealing a good agreement between the SDD and large-scale 2 L bottles [26]. It still needs to be evaluated whether this SDD also reflects thawing at large-scale. Large-scale thawing can affect protein stability. Several studies highlighted significant concentration gradients that evolve during thawing and its negative impact on mAb stability [17–19]. The thawing process is more time-consuming than freezing under similar conditions. Thus, the protein is exposed to an unfavourable environment significantly longer. If the bottle is not homogenised immediately after thawing, this time span extends even further. Until now no SDD is marketed that can adequately mimic the thawing process in widely used rectangular bottles.

While our previous study focused on the validation of the SDD during freezing [26], this study aims to validate the SDD in respect of thawing. Therefore, we compared temperature profiles at several locations in the SDD to a 125 mL and a 2 L bottle. We found significant changes in protein and excipient concentrations in the 2 L bottle that were predicted by the SDD. Excipients play a critical role in ensuring protein stability during freezing and subsequent thawing. Therefore, we did not only compare a model mAb but also the buffer species histidine and the surfactant polysorbate 80 (PS80) at different locations in the SDD to the commercially utilised 2 L bottle. We used these results to assess the density gradient that evolves during thawing and, to our knowledge, has not been examined. Finally, we underline the importance of diffusion, which leads to a rapid equilibration of histidine in the SDD.

## Materials and Methods

### Materials

Polyethersulfone bottle top and syringe filters (0.2 µm) were purchased from VWR International GmbH (Darmstadt, Germany). Cellon S.A. (Bascharage, Luxembourg) provided 2 L and 125 mL PharmaTainer™ polyethylene terephthalate (PET) bottles.

Dipotassium hydrogen phosphate and potassium dihydrogen phosphate needed for the preparation of the mobile phase for the high-performance liquid chromatography (HPLC) were obtained from Merck KGaA (Darmstadt, Germany).

Novartis AG (Basel, Switzerland) provided a 185 mg/mL IgG1 mAb stock solution in a 20 mM histidine buffer at pH 5.5. For the dilution l-histidine monochloride monohydrate and l-histidine were purchased from Merck KGaA (Darmstadt, Germany). Super Refined™ PS80 from Croda International plc (Snaith, UK) was used.

Dulbecco’s phosphate buffered saline 1× (DPBS) without calcium and magnesium chloride, needed for the quantification of PS80, were obtained from Gibco™ (Thermo Fisher Scientific Inc., Waltham, MA, USA). The fluorescence probe 4,4′-dianilino-1,1′-binaphthyl-5,5′-disulfonic acid dipotassium salt (bis-ANS) was purchased from Invitrogen™ (Thermo Fisher Scientific Inc., Waltham, MA, USA).

### Scale-Down Device

**Fig. 1:**
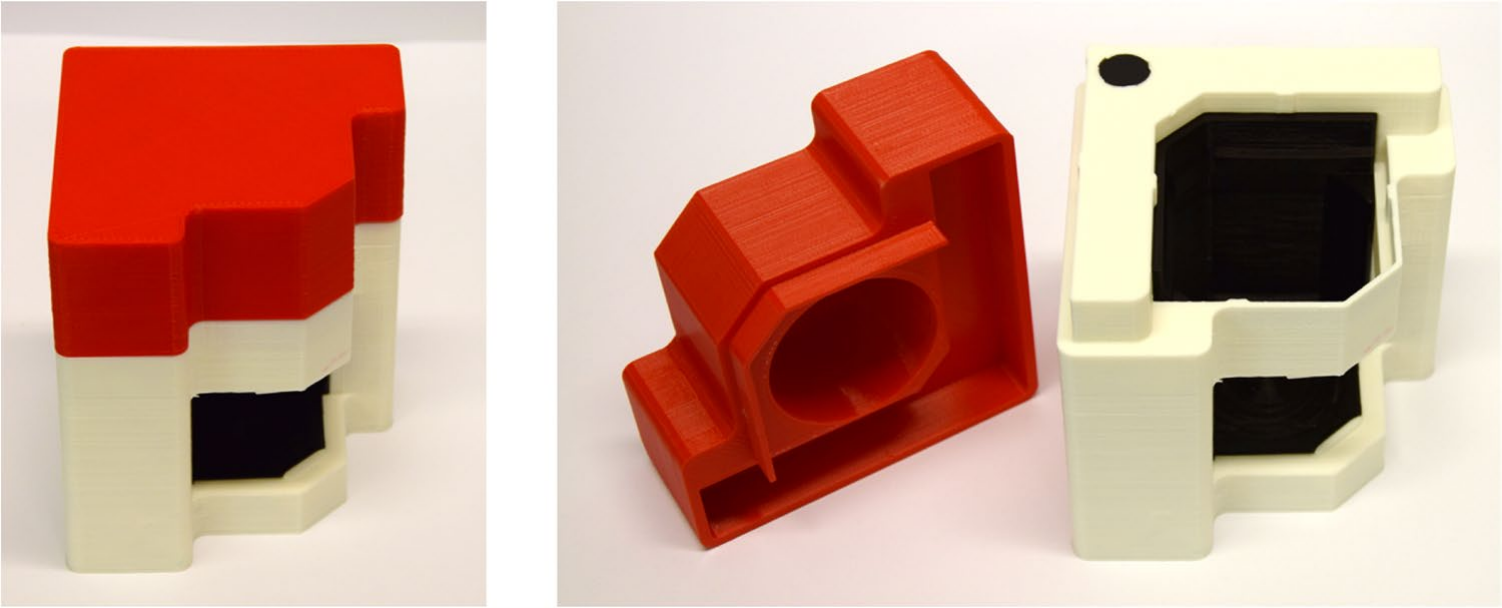
SDD by SmartFreeZ

SmartFreeZ (Porto Salvo, Portugal) provided the SDDs used in this study. A detailed description of the development assisted by CFD can be found elsewhere [26]. The specific SDD was designed to predict thawing in a rectangular 2 L PharmaTainer™ bottle. The 3D printed SDD covers a 125 mL PharmaTainer™ bottle and uses 1% ethanol as a phase change liquid to insulate approximately two walls (Fig. 1). Thereby, a 125 mL bottle shall be utilised to mimic FT processes in a large-scale 2 L bottle. A soft polymer insert prevents circulation of air between the SDD and the bottle. To avoid radiation, a top cover shields the bottle from above. Two SDDs were used simultaneously in back-to-back orientation during measurements. One SDD, filled with highly purified water, was needed for shielding. A second one containing the sample was used for experiments.

### Preparation of Protein Samples

The stock solution was diluted to a final concentration of 5 mg/mL mAb in 20 mM histidine at pH 5.5. The concentration was determined via UV absorption at 280 nm with a NanoDrop One by Thermo Fisher Scientific Inc. (Waltham, MA, USA). Samples containing 0.4 mg/mL PS80 were prepared by spiking a 10 mg/mL PS80 stock solution. All solutions were filtered through a 0.2 µm bottle top or syringe filter. 2 L and 125 mL bottles were 80% filled with 1.6 L and 100 mL, respectively.

### Temperature Mapping During Thawing

**Fig. 2:**
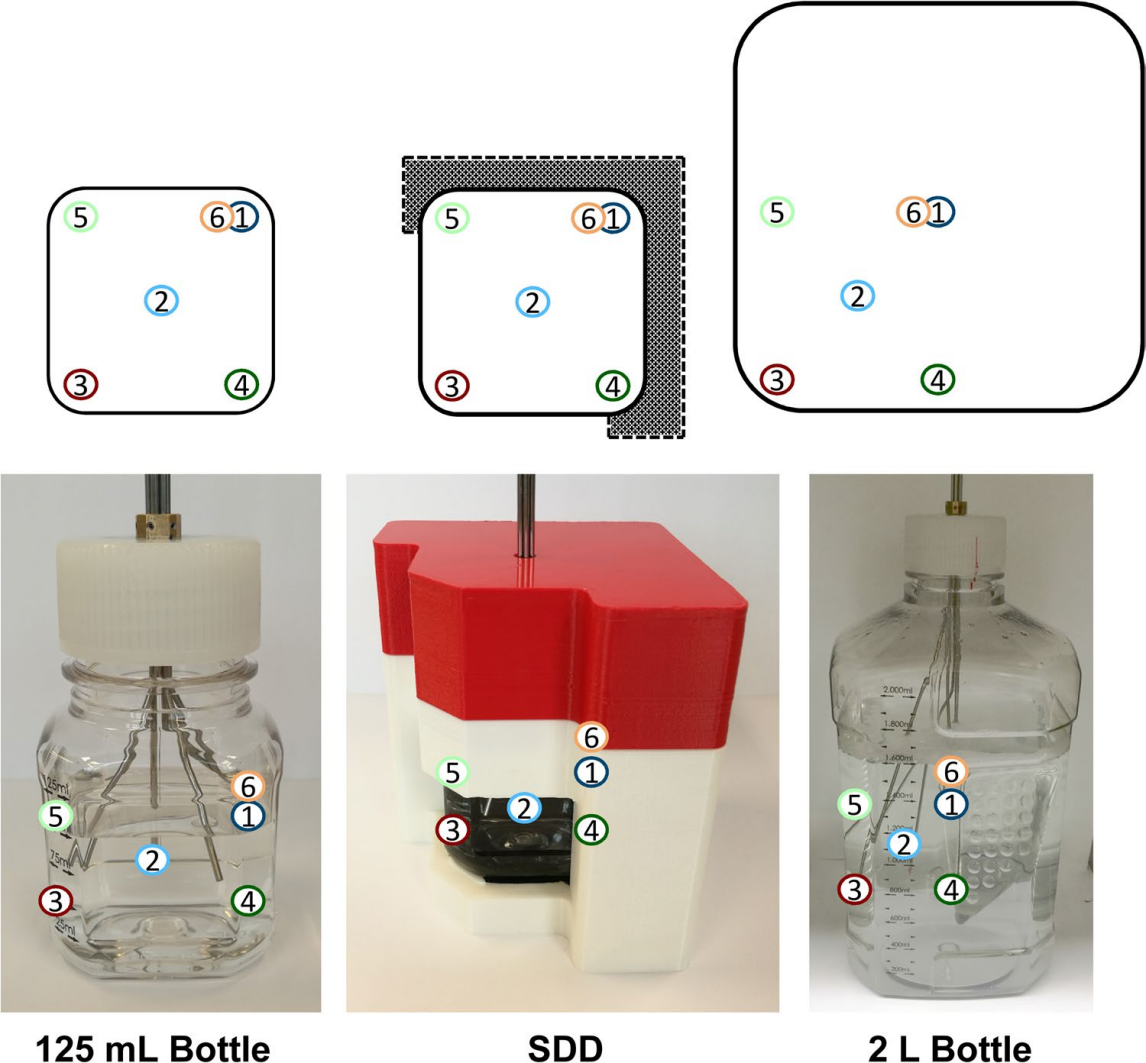
Positioning of TCs in the 125 mL bottle, the SDD and a 2 L PharmaTainer™ bottle (bottom: representative pictures of the positions of the TCs; top: schematic drawing in cross-section from above). Figure adapted from Bluemel et al. [26]

The temperature measurements were performed as previously described [26]. Briefly, five type T thermocouples (TCs) connected to an HH520 handheld data logger thermometer (OMEGA Engineering GmbH, Deckenpfronn, Germany) were positioned at half liquid height in the edges and the centre of the 125 mL bottle using stainless steel capillaries (Acufirm Ernst Kratz GmbH, Berlin, Germany) for reproducible placement (Fig. 2). A sixth TC (TC 6) was placed at the exact position of TC 1 but at 75% liquid level. Six TCs were arranged at equivalent positions in the 2 L bottle. After acclimatisation at 20 °C for 1 h in an MKF 240 air-blast climate chamber (Binder GmbH, Tuttlingen, Germany), the chamber was cooled at maximum rate to − 40 °C and the temperature held for 10 h. Subsequently, the temperature was set to 20 °C with maximum heating rate and the solution thawed until the set temperature was reached at all positions. All temperature measurements were executed in triplicates in independent runs.

### Analysis of Concentration Gradients After Thawing

Concentration gradients after FT were analysed, once in the 2 L bottle and in triplicates in the SDD and the 125 mL bottle. Samples were taken from nine (2 L bottle) or five (SDD and 125 mL bottle) layers. For each layer five 1 mL samples were taken, four in the edges and a fifth from the centre. Samples were taken with a 1 mL serological glass pipette or 1 mL syringes (B. Braun Melsungen AG, Melsungen, Germany) equipped with a Sterican® 0.80 × 120 mm needle (Braun Melsungen AG, Melsungen, Germany).

#### Quantification of mAb and Histidine

Size-exclusion chromatography (SEC) on an Agilent 1200 HPLC with a diode array detector (Agilent Technologies, Santa Clara, CA, USA) allowed the separation and simultaneous quantification of mAb and histidine. Therefore, samples were diluted 1:4 or 1:10 with mobile phase. After centrifugation for 2 min at 25,700×*g* with a Heraeus™ Megafuge™ 16R (Thermo Fisher Scientific Inc., Waltham, MA, USA) 5 µL of each sample were injected. As stationary phase a TSKgel G3000 SWxl column (Tosoh Bioscience GmbH, Griesheim, Germany) and as mobile phase a 150 mM potassium phosphate buffer pH 6.5 at a flow rate of 0.4 mL/min were used. Histidine was quantified at 210 nm and mAb at 280 nm by comparing the areas under the curve to standard curves (R^2^ = 0.9999 and R^2^ = 0.9994).

#### Quantification of PS80

The method for the PS80 quantification was adapted from Zheng et al. [27]. Samples were diluted 1:4 or 1:10 with DPBS and subsequently heated for 5 min at 99 °C. Afterwards, samples were centrifuged for 5 min at 25,700×*g*. 190 µL supernatant were mixed with 10 µL of 1 mM bis-ANS and vortexed for 5 s. 60 µL of each sample were analysed in a Varian Cary Eclipse fluorescence spectrophotometer (Agilent Technologies, Santa Clara, CA, USA) using a quartz cuvette at 380 nm excitation and 500 nm emission with both slits set to 5 nm. A calibration curve of PS80 in DPBS allowed the quantification of PS80 between 0.005 and 0.15 mg/mL (R^2^ = 0.9988).

### Diffusion of Solution Components After Thawing

The diffusion of mAb and histidine after complete thawing of the solution in the SDD was mapped. Samples were taken after complete thawing of the solution (16 h at 20 °C) as well as after additional 24 h and 48 h. To minimize any possible influence of the removed volume on subsequent results, only 0.25 mL were taken per sample. Samples were obtained from the edges in the top layer, the middle layer and at the bottom. Mixing was avoided by tightly attaching the SDD to the grid in the air-blast climate chamber and taking samples slowly with a 1 mL syringe equipped with a needle.

The DynaPro Plate Reader III (Wyatt Technology, Dernbach, Germany) was used to determine diffusion coefficients of mAb and PS80 via dynamic light scattering. Samples with 10 mg/mL mAb or PS80, respectively, were prepared and filtered. 100 µL of each sample was pipetted in triplicates into a 96-well clear bottom plate (Corning Inc., Corning, NY, USA) and ten acquisitions of 5 s at 25 °C taken. The Dynamics V7.8.2.18 software was used for all calculations.

### Analysis of Density Gradients

The changes in density after FT were assessed using a portable density meter DMA 35 Standard (Anton Paar Group AG, Graz, Austria). 15 mL samples were prepared according to concentrations found for each layer during the analysis of the concentration gradients after thawing (Table 1). After a pre-rinse, density was measured in triplicates at room temperature. The density meter extrapolated results to 20 °C.

**Table 1:** Mean concentrations for mAb, histidine and PS80 as determined after thawing in the 2 L bottle, the SDD and the 125 mL bottle

Device	Liquid level indicator [mL]	mAb [mg/mL]	Histidine [mM]	PS80 [mg/mL]
2 L bottle	1600	2.72	12.02	0.24
	1400	3.76	15.38	0.32
	1200	4.19	16.85	0.35
	1000	4.19	16.91	0.34
	800	4.50	18.26	0.36
	600	4.62	18.86	0.37
	400	4.69	19.35	0.38
	200	5.90	28.91	0.50
	Bottom	14.38	52.97	1.10
SDD	100	1.21	6.40	0.11
	75	1.50	7.71	0.13
	50	2.72	15.17	0.23
	25	4.93	30.31	0.39
	Bottom	16.50	44.90	1.32
125 mL bottle	100	3.13	12.70	0.28
	75	3.38	13.56	0.30
	50	4.20	17.57	0.36
	25	5.41	26.35	0.44
	bottom	9.66	38.76	0.86

Corresponding 15 mL samples were prepared to assess changes in density

## Results and Discussion

### Comparison of 3D Temperature Profiles During Thawing

The SDD represents the 2 L bottle by achieving an equivalent cumulative thermal history, although the number of control volumes in the CFD simulations was different. Consequently, temperature measurements at equivalent specific position do not necessarily match. Nonetheless, temperature measurements are important to understand the influence of the SDD during thawing and to characterise the thawing behaviour in comparison to the 2 L and the 125 mL PharmaTainer™ bottles. Within this work, the term thawing time is used and defined as the time needed until a TC, placed inside the bottle, reaches 1 °C after the beginning of heating. At this point in time, ice is completely melted at this location and the intermediate plateau ends as no further heat of melting is needed. The term process time is used to describe the time required to reach 17 °C after the beginning of heating.

**Fig. 3:**
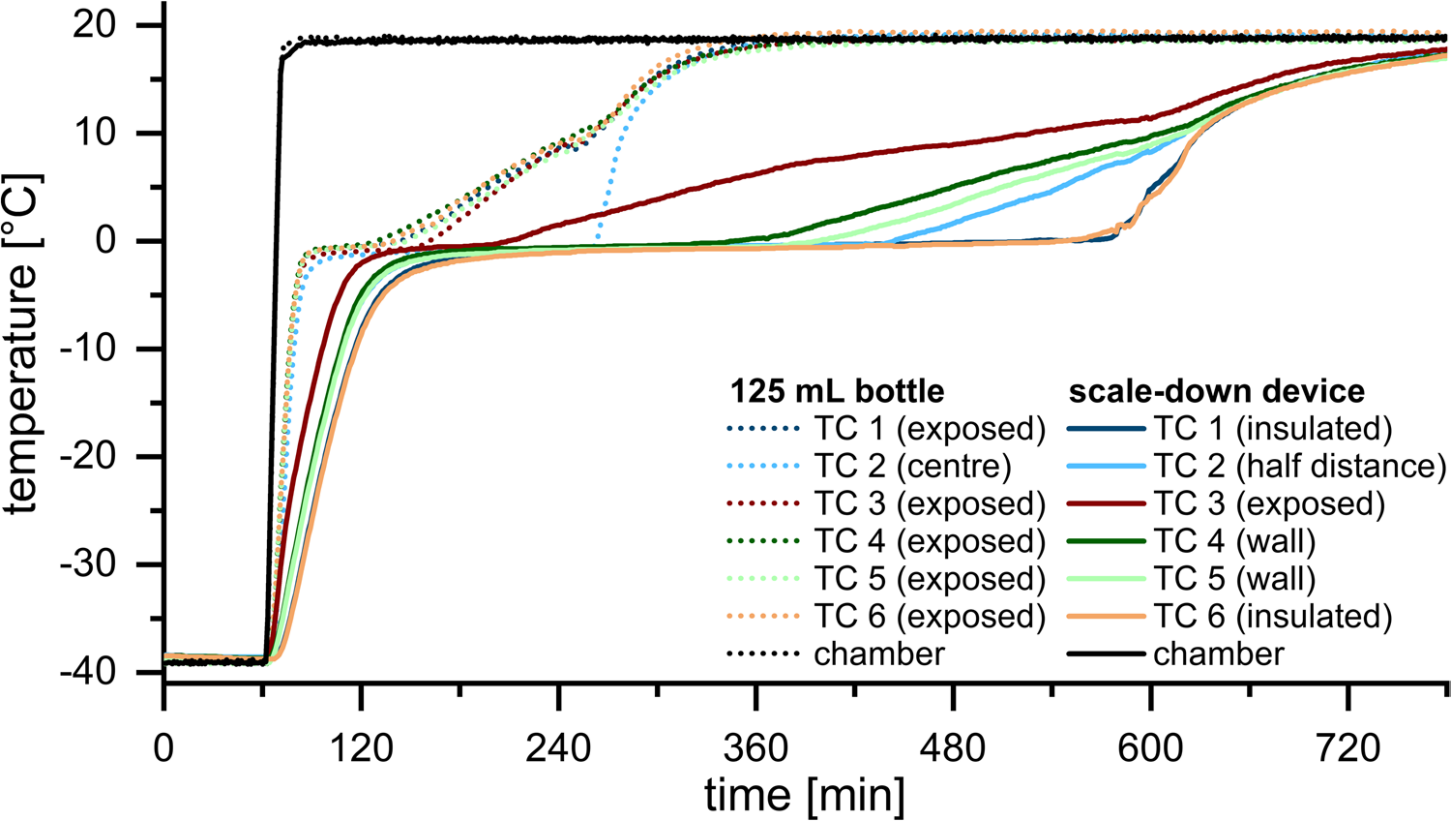
Temperature profiles during thawing in the SDD (solid lines) and the 125 mL bottle (dotted lines)

Temperature profiles during thawing were recorded at six positions. In the 125 mL bottle the TCs in the edges (TC 1 and TC 3–6) recorded similar profiles (Fig. 3). The thawing and process times at these positions were 1.4–1.9 h and 4.1–4.6 h, respectively. The LPTT was the geometrical centre of the bottle (TC 2). Thawing time at this position was 3.4 h, process time was comparable to the TCs in the edges with 4.5 h.

The time needed for thawing was significantly prolonged by the use of the SDD. In addition, the direction of thawing was changed. Thawing started at the exposed edge (TC 3) after 2.8 h and proceeded towards the geometrical centre (TC 2), where the material was thawed after 6.7 h. Similar thawing profiles were recorded for TC 4 and TC 5 near the walls of the SDD with slightly faster thawing within 5.4 h and 6.1 h, respectively. The LPTT was no longer the geometrical centre of the bottle but instead the insulated region (TC 1 and TC 6). After 8.7 h the ice completely melted at both positions. The difference in height between TC 1 and TC 6 did not influence the outcome. The process time was similar for all mapped positions with approximately 12 h.

**Fig. 4:**
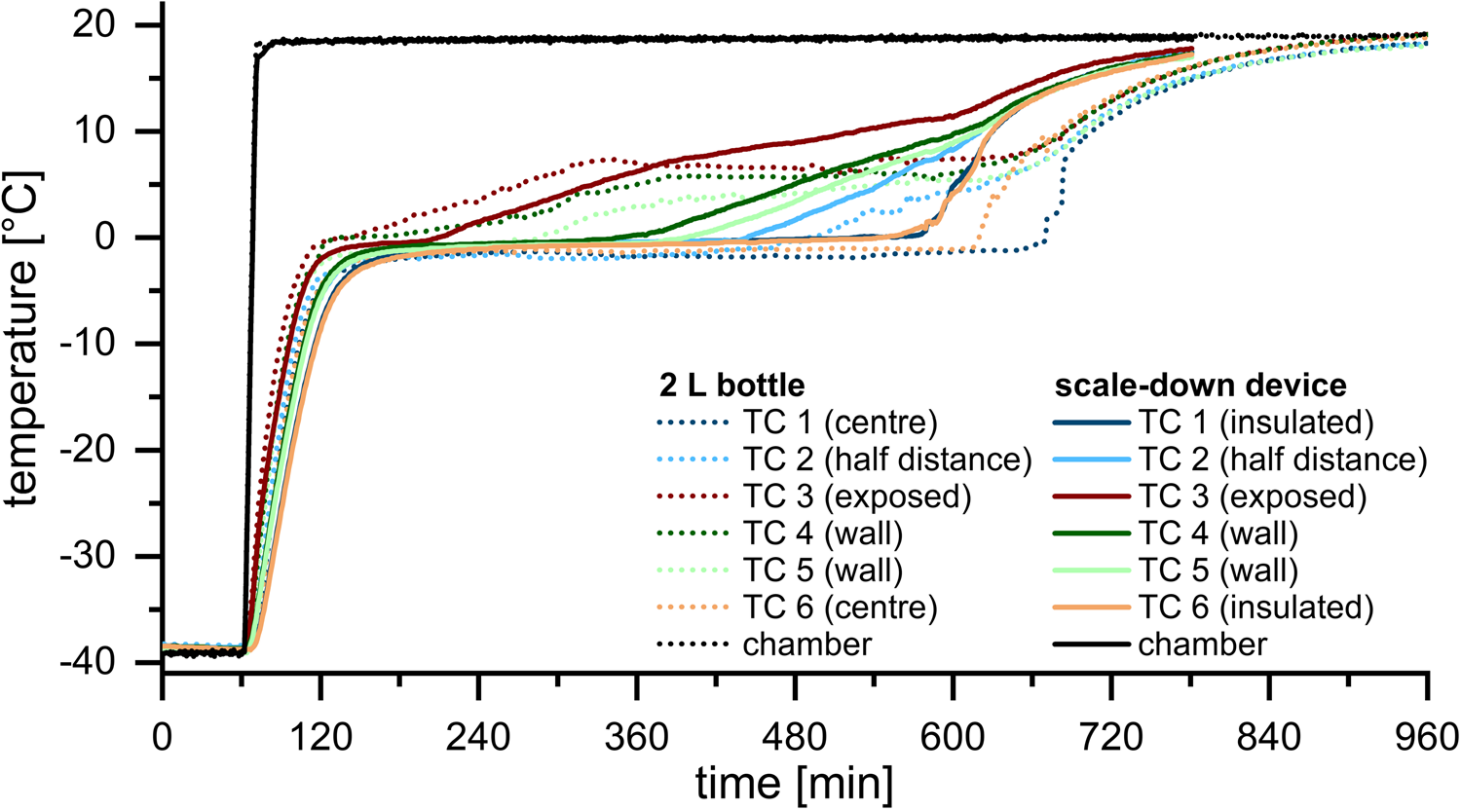
Temperature profiles during thawing in the SDD (solid lines) and the 2 L bottle (dotted lines)

The results for the 2 L bottle in comparison to the SDD are shown in Fig. 4. Thawing started at the exposed edge (TC 3) and took 1.6 h. At the walls thawing needed 2.3 h (TC 4) and 4.0 h (TC 5) and thereby already exceeded the maximum thawing time at the LPTT in the 125 mL bottle. At half height and half distance thawing was completed after 7.2 h (TC 2). The LPTT was the centre of the 2 L bottle, where complete thawing took 9.3 h (TC 6) and 10.0 h (TC 1). In contrast to the SDD, the difference in height between TC 1 and TC 6 changed thawing time by about 40 min. In agreement with the 125 mL bottle and the SDD, temperature profiles at the various locations merged after complete thawing. Therefore, process times were similar for all position with approximately 13 h.

In the 125 mL bottle, thawing started simultaneously at the exposed edges. The walls were heated by the air in the chamber and consequently the edges experienced heat exchange from both adjacent walls. The LPTT was the geometrical centre, the region furthest away from the walls. The latent heat of melting led to a plateau in the observed profiles. As long as the ice was not completely melted, energy was removed from the system to thaw the remaining ice. As soon as no ice was left, the different profiles started to merge. In comparison to the 2 L bottle, thawing time was significantly shorter due to the smaller volume.

The 2 L bottle also started to thaw in the region with highest heat exchange, the exposed edge, followed by the walls. Thawing proceeded towards the geometrical centre. The significantly higher volume increased the time for complete thawing at the LPTT by a factor of three in comparison to the smaller bottle. While the absolute difference in height between TC 1 und TC 6 is small in the SDD and the 125 mL bottle, the higher positioning of TC 6 led to a reduced thawing time in the 2 L bottle. Though more or less stagnant air in the bottle’s headspace provides insulation, heat exchange was still high enough to thaw the bottle also from top towards the centre.

Thawing the same volume in the SDD took significantly longer than in the 125 mL bottle. The SDD provided sufficient insulation to reach adiabatic conditions at two walls. The thawing direction and the heat exchange could be controlled through the exposed window. Consequently, the LPTT was no longer the geometric centre but instead the insulated edge. The temperature profiles were overall similar to those of the 2 L bottle. It took more time to thaw in the exposed regions in the edges and near the walls in the SDD compared to large-scale, whereas there was no difference at half distance. Only slightly less time was needed to completely thaw and fully process the mAb solution in the SDD despite the enormous difference in fill volume by a factor of 16. As the SDD is designed to match the overall thermal history, we expected some regions to thaw slightly faster and others more slowly.

In the SDD thawing was strongly delayed as compared to the 125 mL bottle. Furthermore, the SDD changed the thawing direction so that the LPTT was found in the insulated region and no longer in the geometrical centre. Although thawing and process time were marginally reduced in the SDD, we consider the SDD as representative for the thawing process in large-scale 2 L bottles. In contrast to material consuming large-scale experiments, the SDD needs only a fraction of the volume.

### Comparison of Concentration Gradients After Thawing

Significant changes in concentration throughout the container can be expected after large-scale FT of mAb solutions, potentially affecting mAb stability [17–19]. A screening study revealed marginal relative concentration gradients upon thawing highly concentrated mAb solution. Thus, higher mAb concentrations would level off changes in concentration upon thawing and impede the validation of the SDD. Consequently, we used a diluted solution to validate the SDD under maximally challenging conditions. We quantified the mAb, the buffering agent histidine, and the surfactant PS80 to picture the distribution of the protein versus its stabilisers. Changes in concentration were expressed as the concentration factor (CF), which is the ratio of the concentration of the sample to the initial concentration.

**Fig. 5:**
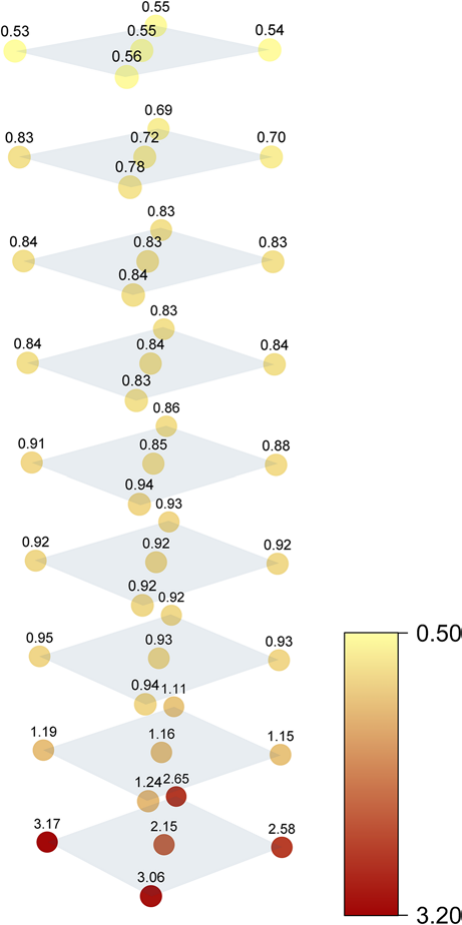
Concentration gradients of the mAb in a 2 L bottle after FT. CF values indicate the change in concentration relatively to the initial mAb concentration of 5 mg/mL

**Fig. 6:**
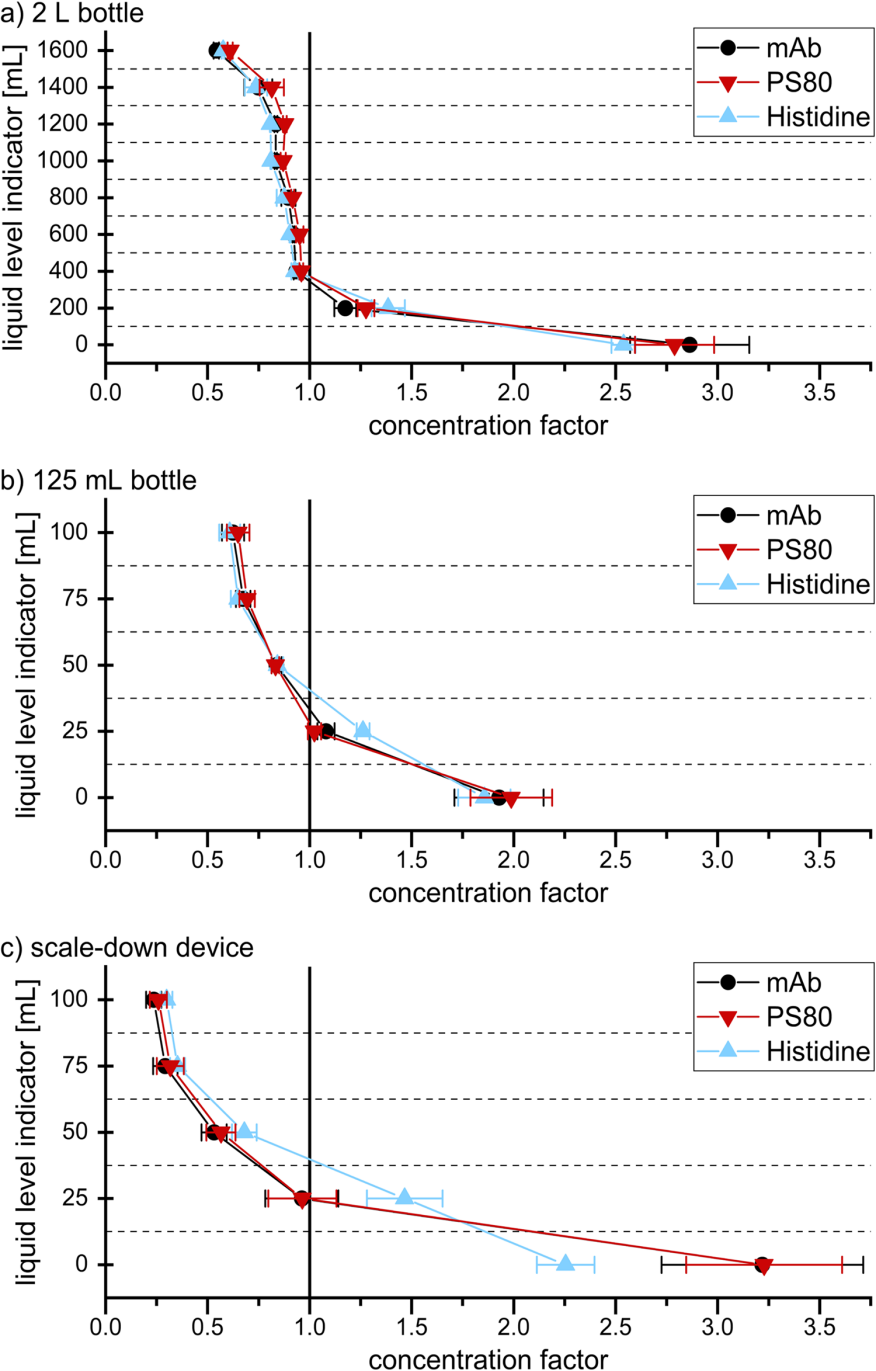
Concentration changes expressed as the CF values after FT of 5 mg/mL mAb, 20 mM histidine and 0.4 mg/mL PS80 in the 2 L bottle, 125 mL bottle and the SDD

For the mAb CF values between 0.53 and 3.17 were found in the 2 L bottle (Fig. 5). These results are in good agreement with previous studies, showing a highly diluted top region and a strongly increased protein concentration at the bottom [18, 19]. The CF steadily decreased with increasing height. In contrast to the three-dimensional cryoconcentration after freezing [26], changes in concentration after thawing can be reduced to a two-dimensional behaviour. CF values were reproducible within each layer. The only exception was the bottom layer with CF values up to 3.17 in the edges, while the centre was significantly less concentrated (CF 2.15). This deviation in the centre is due to the shape of the bottle’s bottom. The bottom is slightly elevated in the centre and reflects a region between the bottom layer and the layer directly above. Because of this deviation, centre samples were excluded from further data evaluation. Figure 6a shows mean and standard deviation of the CF values detected in the edges of the 2 L bottle. The CF values ranged between 0.54 and 2.86 for mAb, 0.61 and 2.79 for PS80 and 0.58 and 2.54 for histidine. All three components behaved similarly in the top and middle region. Near the bottom, maximum mAb and PS80 concentrations were identical. The histidine CF was slightly higher at the 200 mL liquid level and slightly lower at the bottom compared to the mAb and PS80 CF values.

In the 125 mL bottle, CF values between 0.62 and 1.93 for mAb, 0.65 and 1.99 for PS80 and 0.61 and 1.86 for histidine were found (Fig. 6b). For all three components dilution in the top region was only slightly less in comparison to the 2 L bottle. In contrast, the maximum concentrations at the bottom were significantly underestimated. In the second last layer deviations in histidine concentration compared to mAb and PS80 were still noticeable but less pronounced.

CF values between 0.24 and 3.22 for mAb, 0.26 and 3.23 for PS80 and 0.30 and 2.26 for histidine were detected in the SDD (Fig. 6c). As previously shown, mAb and PS80 concentrations changed identically throughout the entity. Near the top, similar CF values for mAb, histidine and PS80 were detected. In contrast, histidine concentration was lower at the bottom and higher at 25 mL liquid level. In comparison to the 125 mL bottle, the SDD had a significant impact on concentration changes. The SDD increased the concentration gradient for all three components. Compared to the 2 L bottle, the dilution in the SDD was enhanced. The maximum mAb and PS80 concentrations at the bottom were insignificantly higher, revealing a good predictive power for this critical region. Deviations between the buffer and the remaining components were emphasized in the SDD.

Maity et al. studied the mechanism of gradient formation during thawing and concluded that the FCM, containing proteins and excipients, melts out of the ice, leaving more or less pure ice behind [19]. Due to its lower density, this ice floats on top and dilutes the top region during melting. At the same time, fractions with higher solute concentrations exhibit increased densities and sink to the bottom of the bottle. The supplementary information illustrates this draining of the FCM out of the ice during thawing of a dyed sucrose solution in a video.

**Fig. 7:**
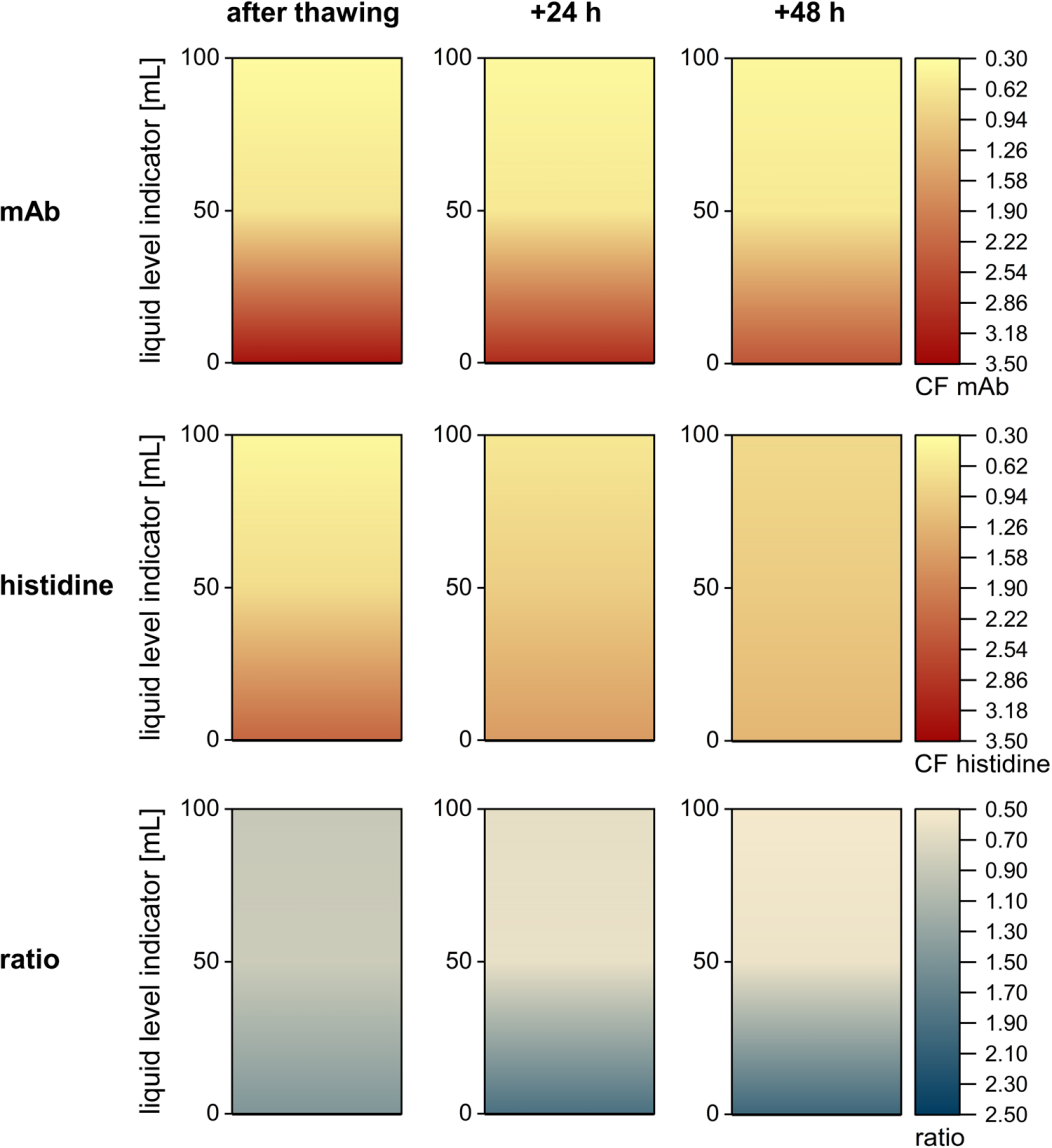
Diffusion of mAb and histidine in the SDD after thawing (16 h) and additional 24 h and 48 h. Colours of the contour plots indicate changes in CF values of mAb and histidine and the mAb to histidine ratio

The difference between histidine CF and both the PS80 and the mAb CF in the second last and the bottom layer may be related to the difference in diffusion between the small histidine molecules and the larger mAb and PS80 micelles during and after thawing. The diffusion coefficients of mAb and PS80 micelles with 4.7 × 10^–11^ m^2^/s and 5.0 × 10^–11^ m^2^/s are significantly lower compared to that of histidine with 7.3 × 10^–10^ m^2^/s [28]. In order to substantiate this effect, we compared the mAb and histidine concentration in the SDD directly after a thawing period of 16 h and an additional 24 h and 48 h storage period at 20 °C (Fig. 7).

After 16 h of thawing a strong concentration gradient was evident for the mAb with CF values between 0.36 and 3.32. This outcome is in good agreement with the results described previously. After additional 24 h and 48 h at 20 °C, the gradient only minimally diminished and showed CF values between 0.39 and 3.00 (+ 24 h) and between 0.41 and 2.49 (+ 48 h), indicating that the significant changes of mAb concentration compared to the initial CF value of 1 (representing 5 mg/mL mAb) sustain for more than 2 days. The dilution of histidine in the top region was similar after 16 h of thawing (CF 0.40), but, as described, the maximum CF was lower (CF 2.26). CF values between 0.60 and 1.58 as well as 0.78 and 1.24 after 24 h and 48 h, respectively, emphasized that the concentration gradient of histidine constantly declined.

The results demonstrate that the larger mAb diffuses much slower into the top region of the SDD than the smaller histidine. This is substantiated by the mAb to histidine ratio. Immediately after thawing, slight differences were already detectable and the ratio progressively shifted towards histidine near the top and towards mAb near the bottom (Fig. 7). In the 125 mL bottle similar diffusion as in the SDD can be assumed. In the larger 2 L bottle, histidine diffusion, as described previously, only became noticeable in the two layers near the bottom due to significantly larger height.

Strong changes in concentration of mAb, histidine and PS80 were detected in the 2 L bottle after thawing with a diluted top region and a concentrated bottom region. Faster diffusion of histidine during and after thawing was the driving force for the different behaviour of mAb and PS80 in comparison to the buffering agent. The 125 mL bottle matched concentrations in the top region, but it significantly underestimated the maximum concentrations at the bottom. This outcome can be deduced from a faster thawing in the smaller bottle. Faster thawing allows less time for the FCM to melt out of the ice. Thus, the ice floating on top incorporates a larger fraction of solutes, resulting in a less diluted top and consequently a less concentrated bottom region. In contrast, the thawing behaviour in the SDD is very similar to the 2 L bottle, enabling the release of the solutes from the ice. The slightly stronger gradient that developed in the SDD reflects a worst-case scenario in respect of changes in concentration after FT in the 2 L bottle.

### Comparison of Density Gradients After Thawing

During thawing the solutes melt out of the ice, leaving ice behind that floats on top [19]. At the same time, fractions with higher solute concentrations and increased density sink to the bottom. Although changes in density are often mentioned as an important factor for cryoconcentration after freezing [9, 18], to our knowledge density gradients after thawing in large-scale bottles have not been analysed yet. Therefore, we assessed density gradients in the 2 L bottle, the SDD and the 125 mL bottle. The large volume that is needed for density measurements would inevitably entail interference and mixing of the different layers in the containers. Consequently, we did not take samples directly from the bottle, but prepared samples according to the concentrations found after thawing in the different devices (see Table 1).

**Fig. 8:**
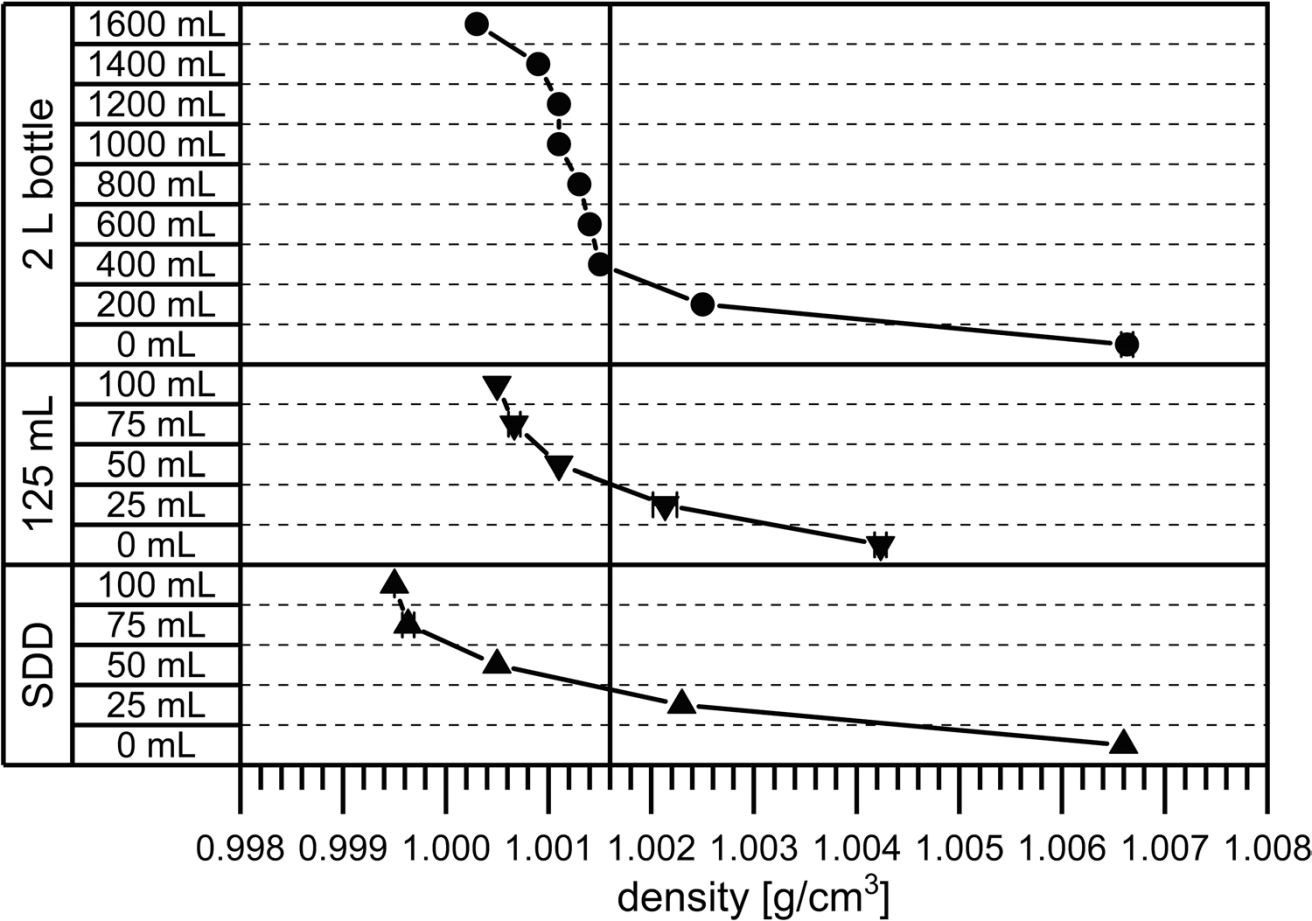
Density gradients in the 2 L bottle, the 125 mL bottle and the SDD. Samples were prepared according to concentrations found in the respective layer indicated by the liquid level indicator. The reference line highlights the initial density of a solution with 5 mg/mL mAb, 20 mM histidine and 0.4 mg/mL PS80

In the 2 L bottle the density steadily increased with each layer from 1.0003 g/cm^−3^ at the top to 1.0066 g/cm^−3^ at the bottom (Fig. 8). In the 125 mL bottle the density was 1.0005 g/cm^−3^ at the top and 1.0042 g/cm^−3^ at the bottom. The density values in the SDD ranged between 0.9995 and 1.0066 g/cm^−3^. The slight density differences between the three systems reflect the differences in the concentrations of the different components. The minimum density in the 2 L bottle was mimicked by the 125 mL bottle, which had a similarly diluted top region. Density near the bottom was markedly underestimated in the 125 mL bottle, reflecting the lower CF values. The SDD showed a more diluted top layer of lower density. Although mAb and PS80 concentrations in the SDD were slightly higher at the bottom, the densities of the bottom layers were identical in the 2 L bottle and the SDD. We assume that the lower histidine concentration balanced the impact of the increased protein and surfactant concentrations in respect of the density.

### Perspective

The SDD used in this study was designed to be universally utilised, regardless of the applied FT process. While our previous study validated the performance of the SDD in respect of freezing [26], the presented study proved the reliability of the SDD during thawing in an air-blast chamber. However, further studies are needed to validate that the SDD can be used regardless of the thawing method.

The design of the specific SDD led to a match of the thermal history compared to a large-scale 2 L bottle. The heat exchange can be adapted by a manipulation of the insulation via adaption of the window of the SDD. SmartFreeZ used this to generate SDDs for rectangular 5 L, 10 L, and 20 L bottles. Studies similar to ours with the 2 L bottle are necessary to proof the reliability of the CFD based concept for the larger bottles.

## Conclusion

In a previous study, we characterised the performance of an innovate SDD during freezing, which requires only a fraction of material in order to mimic and understand the large-scale freezing behaviour of mAb solutions in widely used disposable bottles [26]. In this work, we focused on the validation of the SDD in respect of the subsequently following thawing process of mAb solutions. We compared thawing and process times and quantified concentration gradients of mAb, histidine and PS80 in the SDD to 2 L and 125 mL PharmaTainer™ PET bottles. Furthermore, we assessed changes in density.

Temperature profiles in the 2 L bottle revealed a directed thawing towards the centre of the bottle. LPTT was determined in the geometrical centre with 10.0 h for complete thawing. The 125 mL bottle showed significantly reduced thawing times due to the smaller volume, also progressing towards the centre. The SDD changed the direction of thawing so that no longer the centre, but the insulated edge was the LPTT. Hereby, the overall thawing time was strongly increased to 8.7 h. Thus, we consider the SDD as representative for thawing profiles in large-scale 2 L bottles.

Strong changes in concentration built up during thawing of the large-scale 2 L bottle. The top layer displayed a dilution of approximately 50% compared to the initial mAb, PS80 and histidine concentrations, whereas the concentrations near the bottom increased by a factor of 2.8. The concentration gradients in the 125 mL bottle were significantly smaller for all three components. In contrast, the SDD showed a slightly more pronounced concentration gradient. For mAb and PS80, this gradient persisted for days after thawing due to their slow diffusion. In contrast, the gradient significantly levelled off for the smaller histidine, which can rapidly diffuse into the top region. The density gradients reflected the concentration gradients. The SDD showed an identical density at the bottom and an only slightly lower density near the top compared to the large-scale 2 L bottle. Thus, the SDD can predict thawing processes in large-scale 2 L bottles using only a fraction of the material.

## Supplementary Information

Below is the link to the electronic supplementary material.

Supplementary file1 (MP4 7286 kb)

## Abbreviations

bis-ANS: 4,4′-Dianilino-1,1′-binaphthyl-5,5′-disulfonic acid dipotassium salt
CFD: Computational fluid dynamics
CF: Concentration factor
DPBS: Dulbecco’s phosphate buffered saline
FCM: Freeze-concentrated matrix
FT: Freezing and thawing
HPLC: High-performance liquid chromatography
LPTF: Last point to freeze
LPTT: Last point to thaw
mAb: Monoclonal antibody
PET: Polyethylene terephthalate
PS80: Polysorbate 80
SDD: Scale-down device
SEC: Size-exclusion chromatography
TC: Thermocouple
T_g_′: Glass transition temperature of the maximally freeze-concentrated solution
